# Supplementary material for: Collaborative intelligence in AI: Evaluating the performance of a council of AIs on the USMLE
Source: PLOS Digit Health. 2025 Oct 9;4(10):e0000787. doi: 10.1371/journal.pdig.0000787 (PMC12510544; doi:10.1371/journal.pdig.0000787)
Supplement: S2 File — (ZIP) [file pdig.0000787.s002.zip › S2 Data Raw data files containing unprocessed question inputs and LLM outputs including transcripts of deliberations/STEP3_Question-16-1_Type-MC-NJ.docx]

**Facilitator** **[Round 1]:** The nurse at a local elementary school contacts you about 12 children he has seen in the past 2 weeks. All of the children had similar symptoms of nasal congestion, sneezing, nonproductive coughing, and eye irritation. None of the children had fevers. Several teachers and support staff have reported having the same symptoms. No similar outbreaks have been reported in the community. There was a fire in the school several weeks ago with significant smoke and water damage to classrooms, the cafeteria, and the school ventilation system. Repairs were quickly made and classes resumed. The most likely cause for the students' and teachers' symptoms is exposure to which of the following? (A) Asbestos (B) Legionella pneumophila (C) Mold spores (D) Mycoplasma pneumoniae (E) Respiratory syncytial virus

**AI_B:** Restating the Question: It appears we have a situation where several children from one school along with a number of staff are experiencing common symptoms of nasal congestion, sneezing, coughing and eye irritation without fever. These symptoms developed after a fire that caused substantial damage to the school building and ventilation system. We're tasked with determining the leading probable cause of this outbreak from a listed selection. Planning: The feasibility of each presented cause must be examined. As these symptoms are common in respiratory diseases, it may initially appear reasonable to suspect a pathogen like Legionella pneumophila or Mycoplasma pneumoniae. However, in those cases, we would have normally seen a fever. Similarly, respiratory syncytial virus (RSV) leads to bronchiolitis and is most prevalent in young children and infants, but it usually induces a fever as well. Asbestos, while harmful, tends to provoke severe lung diseases like asbestosis, lung cancer, and mesothelioma, which don't line up with the symptoms. On the other, mold spores can potentially flourish in the damp environment left after the fire and water damage and is capable of producing the symptoms presented. Clinical Reasoning: We need to not rush to infectious causes which, although valid, are not strongly supported by the data available. Taking into consideration that none of the affected persons had fever and the symptoms are localized primarily to the respiratory tract, it is more plausible that we are dealing with an irritant rather than an infectious cause. Asbestos, while harmful, generally produces chronic symptoms over a long course of exposure and leads to severe respiratory conditions that are not consistent with the presented symptoms. The presence of mold, however, fits the scenario quite well. Mold spores grow in moist, dark places and could have developed after extinguishing the fire. Inhaling such spores can trigger an allergic response, leading to the mentioned symptoms: nasal congestion, sneezing, coughing, and eye irritation. Concluding: To synthesize, we can largely disregard the infectious agents (Legionella pneumophila, Mycoplasma pneumoniae, respiratory syncytial virus) due to the absence of fever along with the lack of a documented pandemic in the community, and asbestos due to timeline and symptom discordance. This leaves us with mold spores, and their association with the fire and water damage makes them the most plausible cause. Response: Thus, the most likely cause for the students' and teachers' symptoms is exposure to (C) Mold spores.

**AI_D:** 1) RESTATING THE QUESTION: The nurse at an elementary school is concerned about several students and staff displaying similar symptoms including nasal congestion, sneezing, a non-productive cough, and eye irritation. None of the individuals had a fever. The school had recently undergone repairs after a fire caused significant damage to its infrastructure. Given no similar outbreak in the community is reported, the question is posed concerning the primary cause behind the symptoms, evaluating potential options like exposure to asbestos, Legionella pneumophila, mold spores, Mycoplasma pneumoniae, or the respiratory syncytial virus. 2) PLANNING: The main assumptions in this context are that the symptoms are either due to an infectious or some non-infectious cause. The classroom fire with subsequent smoke and water damage may have led to contamination with either one of the mentioned pathogens or particulate matter like asbestos or mold. Questioning these assumptions, one could argue maybe the children and staff are suffering from cold or allergy that is common but not reported in the community. However, the more plausible assumption clinically is exposure to something within the school premises post the fire-related damage. 3) CLINICAL REASONING: The symptoms being experienced by the children and staff point to a mix of respiratory and ocular irritation (nasal congestion, sneezing, nonproductive coughing, and eye irritation). These symptoms are commonly associated with allergenic or irritant exposure rather than a specific infection, especially in the absence of fever. Considering the various options: (A) Asbestos primarily causes long-term consequences like asbestosis, lung cancer and mesothelioma, it wouldn't feasibly lead to these acute symptoms. (B) Legionella pneumophila causes Legionnaires' disease, presenting with a high fever, which is absent in these cases. (D) Mycoplasma pneumoniae and (E) respiratory syncytial virus primarily cause bronchitis and pneumonia, also typically associated with a fever. (C) Mold spores becomes a more plausible assumption. Mold growth is a common issue in water-damaged buildings. Exposure to mold can provoke various health issues, particularly respiratory symptoms, which align with the observed symptomatology. 4) CONCLUDING: To connect the dots using the above reasoning, it would be likely that the fire damage led to water infiltration and conducive conditions for mold growth. The subsequent exposure to these mold spores could plausibly cause the symptoms seen in the children and support staff. 5) RESPONSE: Given the overlay of presented symptoms, history of water and smoke damage due to fire at the school, absence of fever and similar symptoms in a diverse group of individuals, the most likely cause of their symptoms is exposure to mold spores (Choice C).

**AI_A:** Restating the question would be something like: "The local elementary school's nurse has contacted me about 12 students over the last couple of weeks showing symptoms of nasal congestion, sneezing, nonproductive cough, and eye irritation. None have fevers. Even teachers and other staff have reported to have similar symptoms. There have been no such outbreaks elsewhere in the community, and there had been a recent fire incident causing critical smoke and water damage to parts of the school including the ventilation system, which has since been repaired. We need to identify the possible source or cause behind these symptoms, taking into account the options provided: (a) Asbestos, (b) Legionella pneumophila, (c) Mold spores, (d) Mycoplasma pneumoniae, or (e) Respiratory syncytial virus." To plan the solution for this, one would need to question each option, and prioritize them likely to what may have been present in the school environment. (a) Asbestos could possibly cause respiratory symptoms, however, it generally leads to chronic illnesses like mesothelioma and not acute symptoms as seen here. (b) Legionella pneumophila infection, also known as Legionnaires' disease often leads to pneumonia with associated high fever, muscle pain, and sometimes diarrhoea, which is not present here. (c) Mold spores can indeed cause the observed symptoms if inhaled in large amounts, and it might as well proliferate in moist environments after the fire and water damage tailored for that. (d) Mycoplasma pneumoniae causes walking pneumonia and is usually associated with a low-grade fever, malaise, and an onset of a few weeks, which doesn't really align with the symptomatology here. (e) Respiratory syncytial virus is a common cause of bronchiolitis and pneumonia in children, usually associated with a fever, which is not the case here. Connecting the clinical reasoning forward, asbestos and Legionella pneumophila are less probable because they cause chronic and severe symptoms respectively. Mycoplasma pneumoniae and the respiratory syncytial virus both usually are associated with fevers and other systemic symptoms that are not present in this case. Therefore, the most likely cause appears to be mold spores, which can cause respiratory and hypersensitivity reactions, similar to the symptoms experienced by the children and staff. After the fire, immediate repairs would have been done and the classes resumed, but the moist environment following the water damage could have been an ideal habitat for the growth of mold. These spores could have then dispersed through the school's ventilation system, causing widespread exposure among the school's population. So by connecting reasoning and solution as per the mentioned process, the most likely cause of the symptoms experienced by the students and the staff might be exposure to mold spores (Option C). This fits with the respiratory and ocular symptoms experienced, the lack of similar symptoms in the wider community, the recent fire and water-related history in the school and the subsequent likely incubation environment for molds.

**AI_E:** RESTATING THE QUESTION: The local school nurse has shared a concern regarding 12 students who have been presenting with similar symptoms such as nasal congestion, sneezing, nonproductive cough, and eye irritation over the past couple of weeks. Interestingly, none of the affected children exhibited fever. Several staff members have reported similar symptoms. It's important to note that no comparable outbreaks have been registered in the wider community. Additionally, the school experienced a fire a few weeks back leading to significant smoke and water damage, primarily in the classrooms, cafeteria, and school ventilation system. Repairs were instituted promptly and school activities resumed. Given this scenario, the query is about identifying the most likely cause of the students' and teachers' symptoms from five options: asbestos, Legionella pneumophila, mold spores, Mycoplasma pneumoniae, or Respiratory syncytial virus. PLANNING: When addressing the cause of the symptoms, it would be critical to consider the recent fire at the school, the subsequent repairs, and the resumption of classes. It could be assumed that the cause is related to something introduced into the environment (e.g., asbestos or mold) or an infection resulting from a compromised ventilation system possibly housing a pathogen (e.g., L. pneumophila). Alternatively, Mycoplasma pneumoniae and Respiratory syncytial virus could cause similar symptoms; however, the lack of fever and absence of community outbreak leads to questioning the likelihood of this assumption. Therefore, these infectious causes should be lower on our priority list. CLINICAL REASONING: Asbestos, a fibrous mineral used in construction, causes long-term diseases such as mesothelioma and asbestosis which typically present with symptoms like shortness of breath and cough, all non-acute. Legionella pneumophila, is known to cause Legionnaires' disease, a type of pneumonia that would likely involve fever and community outbreak, so it seems unlikely. Mycoplasma pneumoniae, a common cause of community-acquired pneumonia, often presents with fever and respiratory symptoms, but there was no temperature recorded in this case, making it less likely. The Respiratory syncytial virus causes lower respiratory infections, primarily in young children. Given the symptoms' absence of fever, and the prior school fire with the water damage, mold spores resulting from the water damage associated with the fire emerge as a consistent and reasonable clinical likelihood. CONCLUDING: If the temporal relationship between the fire, the incidence of the symptoms, the type of damage (smoke and particularly water), and the location of the damage (ventilation system, classrooms) are all taken into account, it appears that mold spores could have been given an environment to flourish and potentially be dispersed by the ventilation system. This could expose both children and adults to the spores, triggering these symptoms. The symptoms experienced are common reactions to mold exposure, making it a strong candidate out of the provided options. RESPONSE: Based on the evidence available and the process of elimination, it would appear that the most likely cause for the students' and teachers' symptoms is exposure to option (C) Mold spores.

**AI_C:** (1) RESTATING THE QUESTION: The query is about determining the most probable cause of the symptoms, namely nasal congestion, sneezing, nonproductive coughing, and eye irritation, manifesting among twelve children and several staff members at a local elementary school. These symptoms have emerged following a fire which resulted in significant smoke and water damage in a school, impacting classrooms, the cafeteria, and the ventilation system. Besides the school, no similar outbreaks have been reported in the surrounding community. Fever was not present in the affected children. Here is a quick overview of the potential culprits: Asbestos - A natural mineral widely used in many industries due to its heat tolerance and fireproof properties. It can cause issues when its tiny fibers become airborne and are breathed in. Legionella pneumophila - A bacterium causing Legionnaires' disease, a severe form of pneumonia. Mold spores - Fungi that spread by forming and dispersing tiny particles known as spores. They could grow at damp places. Mycoplasma pneumoniae - A bacterium causing mild cases of pneumonia, known as "walking pneumonia." Respiratory syncytial virus - A virus causing infections of the lungs and respiratory tract, fairly common in children. (2) PLANNING: The symptoms' sudden onset in a group of children and staff following the fire incident prompts the assumption of an external/environmental factor introduced to the school grounds. Since the principal symptoms relate to respiratory and ocular irritation and there are no fever signs, an infectious agent like Legionella pneumophila, Mycoplasma pneumoniae, or Respiratory syncytial virus is less probable. These organisms typically induce febrile responses. Moreover, given that no similar outbreaks have been reported elsewhere in the community, this situation suggests a localized issue within the school building. Hence, attention turns to environmental factors, such as Asbestos or Mold spores, resulting from fire reconstruction or post-fire conditions. (3) CLINICAL REASONING: Restoring a fire-ravaged structure, particularly the ventilation system, in a hurried manner might leave room for overlooked problems, potentially prompting a hazardous environment. Thus, the proliferation of mold spores, typically following water damage, and asbestos presence, commonly found in construction materials, become crucial considerations. However, asbestos exposure symptoms would likely take longer to manifest, often years after exposure, and typically result in mesothelioma, asbestosis, or lung cancer, distinct from the symptoms observed. In contrast, exposure to mold spores often generates symptoms consistent with the case presented—nasal congestion, eye irritation, coughing, and sneezing—especially if the individuals affected are in an enclosed setting with poor ventilation. Thus, mold spores exposure should be clinically prioritized. (4) CONCLUDING: By considering the nature of the school environment post-fire, the likelihood of the quick restoration process neglecting areas of potential hazard, the absence of fever in the affected individuals, and the fact that similar outbreaks have not been witnessed in the surrounding community, it becomes clear that exposure to Mold spores is highly probable. (5) RESPONSE: The symptoms presented by students and teachers at the school are most likely due to exposure to Mold spores (C). The damp conditions caused by water damage, in combination with the fast, possibly substandard repairs, could have created an environment conducive for mold growth, particularly in the ventilation system. Mold spore exposure can result in the respiratory and ocular irritation noted in the children and staff.

**Facilitator** Restating the question, the local elementary school's nurse has reported that 12 students and several staff members have been experiencing symptoms of nasal congestion, sneezing, nonproductive cough, and eye irritation over the past few weeks. None of the affected individuals have fevers. This situation arose after a fire incident at the school, which caused significant smoke and water damage, particularly to the ventilation system. The school was promptly repaired and classes resumed. No similar outbreaks have been reported in the wider community. The task is to identify the most likely cause of these symptoms from the given options: Asbestos, Legionella pneumophila, Mold spores, Mycoplasma pneumoniae, or Respiratory syncytial virus. Planning the solution, it's crucial to evaluate each option in relation to the school environment and the symptoms presented. Asbestos, while harmful, typically leads to chronic illnesses like mesothelioma, which doesn't align with the acute symptoms observed. Legionella pneumophila, the cause of Legionnaires' disease, often leads to pneumonia with associated high fever, which is not present in this case. Mycoplasma pneumoniae and the respiratory syncytial virus both usually cause conditions associated with fever and other systemic symptoms that are not present in this case. The clinical reasoning points towards mold spores as the most likely cause. Mold spores can proliferate in moist environments, such as the one created by the water damage from the fire. Exposure to mold spores can cause respiratory and hypersensitivity reactions, similar to the symptoms experienced by the children and staff. The fact that no similar outbreaks have been reported in the community further supports the idea that the cause is localized within the school building, possibly in the ventilation system. Concluding, the most likely cause of the symptoms experienced by the students and staff is exposure to mold spores. This conclusion is based on the nature of the symptoms, the absence of fever, the recent history of fire and water damage at the school, and the lack of similar symptoms in the wider community. Therefore, the response to the prompt is that the most likely cause for the students' and teachers' symptoms is exposure to (C) Mold spores.
